# Defatting and thinning full-thickness skin grafts using a rolled elastic bandage and stabilizing syringe needles

**DOI:** 10.1016/j.jdin.2025.09.001

**Published:** 2025-09-25

**Authors:** Hyo Jin Yoon, Jun Young Kim

**Affiliations:** Department of Dermatology, School of Medicine, Kyungpook National University, Kyungpook National University Hospital, Daegu, South Korea

**Keywords:** defatting, elastic bandage, full-thickness skin grafts, syringe needles

## Surgical challenge

Defatting and thinning of full-thickness skin grafts are typically performed by placing the graft donor on the surgeon’s fingers or the back of the hand.^1^ However, when placed on the fingers, the varying hardness of the finger’s skin makes it difficult to achieve a uniform thickness, requiring significant time and increasing the risk of creating holes in the graft. When placed on the back of the hand, securing the graft becomes challenging because 1 hand is occupied. The use of a rolled towel and staples for graft stabilization requires manual preparation, and the staples are difficult to reposition once applied.^1^

## Solution

**Fig 1:**
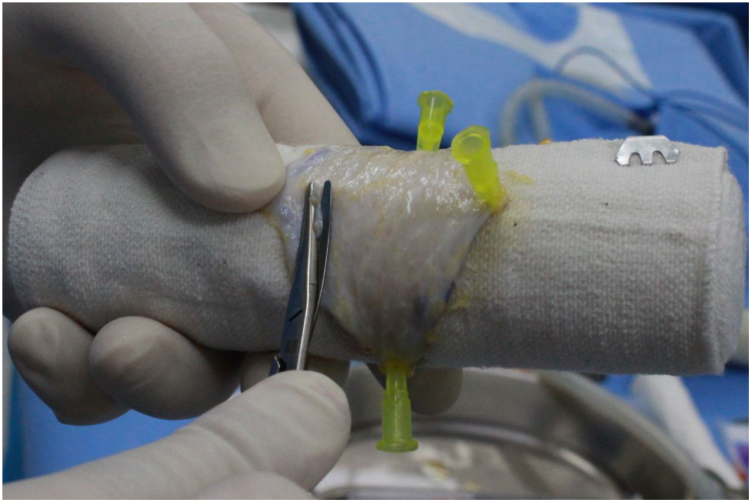
Application of elastic bandage for defatting and thinning of full-thickness skin grafts. A full-thickness skin graft measuring 7.5 × 4 cm was harvested from the right inguinal region for the reconstruction of a defect following the excision of an acral melanoma on the left sole. After removing the cover of a 6-inch-wide elastic bandage, the tightly rolled bandage was used without unrolling it. The full-thickness skin graft was spread perpendicularly to the long axis of the bandage. Three syringe needles were then used to secure the graft at 3 peripheral points. While holding the bandage with the left hand and stabilizing the graft with the left thumb, defatting and thinning were performed using Metzenbaum scissors. Applying consistent pressure ensures effective defatting and thinning of the graft. Even if there are variations in the applied pressure while using Metzenbaum scissors, the bandage acts as a cushion, preventing unintended perforations. The elastic bandage serves as an excellent support platform during the procedure.

Using a 6-inch-wide elastic bandage as a support for defatting and thinning during full-thickness skin graft preparation offers a convenient and effective approach. The elastic bandage is prerolled tightly, allowing for immediate use by simply removing the cover. Because the typical graft donor size in dermatologic surgeries is smaller than 10 × 10 cm, the bandage provides sufficient surface area to spread and stabilize the graft. Additionally, the excess portion of the bandage can be easily held, making handling more convenient (Fig 1).

**Fig 2:**
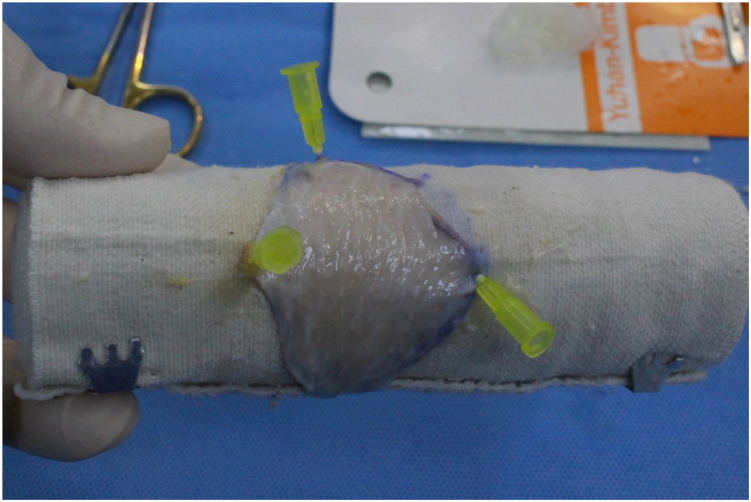
A uniformly thinned graft using an elastic bandage and adjustable needle fixation. If the syringe needles interfere with the defatting and thinning process, their positions can be adjusted accordingly. This approach allows for complete and uniform defatting and thinning, resulting in a full-thickness skin graft with consistent thickness across all areas.

To secure the graft, 3 peripheral points of the graft are fixed using syringe needles. When the graft is securely and evenly spread on the elastic bandage, it becomes nearly impossible to create unintended perforations with Metzenbaum scissors. By applying appropriate pressure during defatting and thinning, a uniformly thinned graft can be achieved with precision (Fig 2). To prevent the needles from obstructing the defatting process, their positions can be adjusted as needed.

This method offers several advantages. The elastic bandage is premanufactured, requires no special preparation, and is appropriately sized for most dermatologic full-thickness skin grafts. It can also be employed in cases where blue towels or staples are not used. Needle fixation provides stable support while permitting easy repositioning or removal, thereby facilitating uniform defatting and thinning of the graft. Moreover, the technique is straightforward and easily applicable, making it suitable even for beginners.

## Conflicts of interest

None disclosed.
